# Chronological Age Prediction: Developmental Evaluation of DNA Methylation-Based Machine Learning Models

**DOI:** 10.3389/fbioe.2021.819991

**Published:** 2022-01-24

**Authors:** Haoliang Fan, Qiqian Xie, Zheng Zhang, Junhao Wang, Xuncai Chen, Pingming Qiu

**Affiliations:** Guangzhou Key Laboratory of Forensic Multi-Omics for Precision Identification, School of Forensic Medicine, Southern Medical University, Guangzhou, China

**Keywords:** DNA methylation, CpG, chronological age prediction, machine learning, stepwise regression, support vector regression, random forest regression, epigenetic clock

## Abstract

Epigenetic clock, a highly accurate age estimator based on DNA methylation (DNAm) level, is the basis for predicting mortality/morbidity and elucidating the molecular mechanism of aging, which is of great significance in forensics, justice, and social life. Herein, we integrated machine learning (ML) algorithms to construct blood epigenetic clock in Southern Han Chinese (CHS) for chronological age prediction. The correlation coefficient (*r*) meta-analyses of 7,084 individuals were firstly implemented to select five genes (*ELOVL2*, *C1orf132*, *TRIM59*, *FHL2*, and *KLF14*) from a candidate set of nine age-associated DNAm biomarkers. The DNAm-based profiles of the CHS cohort (240 blood samples differing in age from 1 to 81 years) were generated by the bisulfite targeted amplicon pyrosequencing (BTA-pseq) from 34 cytosine-phosphate-guanine sites (CpGs) of five selected genes, revealing that the methylation levels at different CpGs exhibit population specificity. Furthermore, we established and evaluated four chronological age prediction models using distinct ML algorithms: stepwise regression (SR), support vector regression (SVR-eps and SVR-nu), and random forest regression (RFR). The median absolute deviation (*MAD*) values increased with chronological age, especially in the 61–81 age category. No apparent gender effect was found in different ML models of the CHS cohort (all *p* > 0.05). The *MAD* values were 2.97, 2.22, 2.19, and 1.29 years for SR, SVR-eps, SVR-nu, and RFR in the CHS cohort, respectively. Eventually, compared to the *MAD* range of the meta cohort (2.53–5.07 years), a promising RFR model (*ntree* = 500 and *mtry* = 8) was optimized with an *MAD* of 1.15 years in the 1–60 age categories of the CHS cohort, which could be regarded as a robust epigenetic clock in blood for age-related issues.

## Introduction

Aging is an inevitable, universal and natural phenomenon that occurs with age, characterized by progressive decline in organismal function and more susceptible to irreversible degenerative disease and even death (Sen et al., 2016). Accumulating studies have linked aging to epigenetic alterations (Grönniger et al., 2010; Sen et al., 2016; Horvath and Raj, 2018). As such, aging denotes an elementary epigenetic phenomenon, and epigenetic changes are widely considered to play a crucial role in aging (Fraga et al., 2005; Boks et al., 2009). Epigenetics is often defined by changes in gene function that do not involve any changes in DNA sequence, and epigenetic changes during aging mainly include histone modification and DNA methylation (DNAm) (Parson, 2018; Unnikrishnan et al., 2019).

DNAm is a chemical modification that mainly occurs in cytosine-phosphate-guanine (CpG) loci, especially in the CpG islands. In fact, an initial study of age-associated methylation in normal tissue was motivated by the study of methylation in cancer (Esteller, 2002). Cancer is well recognized as a disease of aging. For example, Christensen et al. verified this by proposing that variations in age- and exposure-related methylation may significantly contribute to increased susceptibility to several diseases (Christensen et al., 2009). Emerging studies are beginning to work on the associations between methylation profiles and human tissues; however, most of them have focused on therapeutic targets for pathological tissues (Suzuki et al., 2006; Portela and Esteller, 2010; Gao et al., 2019).

In forensics, DNAm biomarkers mainly focus on normal tissues, and employing methylation levels of strongly age-related CpGs (AR-CpGs) into construction of age predictive models has become a mainstream of age-estimation strategies (i.e., epigenetic clock) (Horvath and Raj, 2018). Epigenetic clock, which measures alterations in specific CpGs, is a synonym of a highly accurate age estimator based on DNAm levels (Unnikrishnan et al., 2019). As the most promising molecular age estimator, epigenetic clock can not only accurately predict age, mortality, or morbidity but also help to disentangle the role of DNAm in the mechanisms of aging, therefore facilitating anti-aging interventions (Jylhävä et al., 2017; Horvath and Raj, 2018; Unnikrishnan et al., 2019). Moreover, the epigenetic clocks can be utilized in other non-clinical areas, such as 1) forensic DNA phenotyping, including scenes in criminal investigation or catastrophic disaster (Gršković et al., 2013; Vidaki et al., 2013; Parson, 2018); 2) potentially determination of age of criminal responsibility for judgement (Gršković et al., 2013); and 3) children and youth growth monitoring, athlete selection, and social welfare recognition in our social life (Weidner et al., 2014).

To date, even though the relationship between aging and CpG methylation is complicated (Tra et al., 2002), large series of AR-CpGs were applicable for age prediction from methylation analysis, and quite a few epigenetic clocks of different populations were generated, providing references for distinct forensic scenarios. For example, Hannum et al. (2013) identified 71 AR-CpGs using the Illumina Infinium HumanMethylation450 BeadChip assay and built an age calculator with a correlation of 96% and a median absolute deviation (*MAD*) value of 3.9 years. Naue et al. chose 15 AR-CpGs for methylation analysis using the massive parallel sequencing method and proposed a regression model with an *MAD* value of 3.21 years (Naue et al., 2017). Smeers et al. investigated 16 AR-CpGs by pyrosequencing method and constructed three statistical prediction models with *MAD* values of 3.21, 3.20, and 3.26 years, respectively (Smeers et al., 2018). Dias et al. tested 5 AR-CpGs using the multiplex SNaPshot assay and developed an age prediction model based on 4 of them, with an *MAD* value of 4.97, which explains 92.5% variation in age (Dias et al., 2020).

As mentioned above, the *MAD* values for most DNAm-based age prediction models were more than 3 years (Zbieć-Piekarska et al., 2015b; Cho et al., 2017; Naue et al., 2017; Vidaki et al., 2017; Aliferi et al., 2018; Smeers et al., 2018; Dias et al., 2020), and also many factors have influences on age prediction accuracy, which limited its practical application. For example, different human body fluids (blood, semen, saliva, etc.) exhibit distinct methylation patterns (Jung et al., 2019), and in different populations/genders, the same DNAm biomarkers show diverse methylation levels in the same age category (Zbieć-Piekarska et al., 2015b; Cho et al., 2017; Dias et al., 2020). In addition, there are various alternative approaches (genome-wide DNAm, Illumina BeadChip, bisulfite pyrosequencing, etc.) for DNAm detection, while the bisulfite targeted amplicon pyrosequencing (BTA-pseq) technology supports standardized and cost-effective high-throughput analysis, which is generally relatively accurate. Except for the selection of population-/gender-/tissue-specific DNAm biomarkers and detection methods, the algorithm also has an impact on the age-prediction accuracy. Aliferi et al. (2018) compared the efficiency of 17 machine learning (ML) models based on the same MPS data and suggested that multiple linear regression (MLR) models did not outperform the generalized regression neural network (GRNN) model and several non-linear approaches showed increased accuracy, especially for support vector machine polynomial (SVMp). Xu et al. (2015) found that the *MAD* values reduced in the models of nonlinear regression, BP neural network, and support vector regression (SVR) by using the same CpGs when comparing with the MLR model. Garali et al. compared six different statistical models with the MLR model of Zbiec-Pierkarska (Zbieć-Piekarska et al., 2015b), and the results suggested that multiple quadratic regression (MQR), SVM, gradient boosting regressor (GBR), and MissMDA (mMDA) models outperformed the MLR model for age prediction from *ELOVL2* (Garali et al., 2020).

Hence, in order to establish robust age prediction ML models for Southern Han Chinese (CHS), a candidate set of nine DNAm biomarkers was collected by meta-analyses of 7,084 individuals. Among them, five promising age-related genes (34 CpGs) were selected according to the correlation coefficient (*r*) ranking and Gene Expression Omnibus (GEO) data mining by AgeGuess (Gao et al., 2020). The DNAm-based profiles of the CHS cohort (240 blood samples with ages of 1–81 years) were generated by BTA-pseq. In addition, four different ML algorithms, stepwise regression (SR), SVR (including eps- and nu-regression), and random forest regression (RFR), were used to establish the age-prediction models based on AR-CpGs (*|r|*≥0.7). The samples were randomly divided into different datasets according to different genders and chronological ages, and we evaluated the model efficiencies in Training and Validation sets by *MAD* and root mean square error (*RMSE*) values, to find the best-performing ML model of CHS to estimate the chronological ages in practice.

## Materials and Methods

### AR-CpG Selection and Sample Collection

**FIGURE 1:**
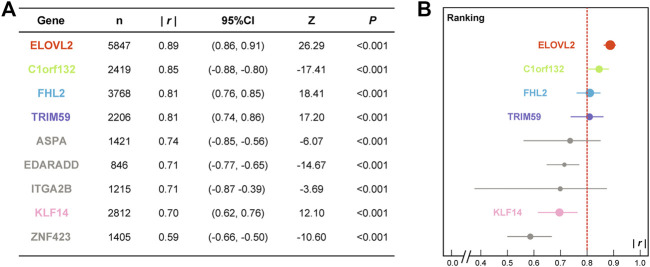
Detailed meta-analysis results **(A)** and correlation coefficient ranking **(B)** of the candidate age-associated gene set. (*n*, sample size; *|r|*, absolute value of correlation coefficient; CI, confidence interval; *p*, significance of Z test.)

The bibliographic search strategies were developed according to the DNAm-based age prediction studies with *MAD* values less than 5 years between 2014 and 2021, and we collected a cohort of 7,084 individuals from 16 countries or populations (Weidner et al., 2014; Bekaert et al., 2015; Xu et al., 2015; Zbieć-Piekarska et al., 2015a; Zbieć-Piekarska, et al., 2015b; Park et al., 2016; Zubakov et al., 2016; Cho et al., 2017; Feng et al., 2018; Alsaleh and Haddrill, 2019; Daunay et al., 2019; Jung et al., 2019; Li et al., 2019; Dias et al., 2020; Garali et al., 2020; Lau and Fung, 2020; Pan et al., 2020; Piniewska-Róg et al., 2021; Sukawutthiya et al., 2021; Woźniak et al., 2021; Xiao et al., 2021). The correlation coefficient (*r*) ranking of nine age-associated genes was obtained by meta-analyses (Figure 1 and Supplementary Figure S1). We selected four promising DNAm biomarkers (*ELOVL2*, *C1orf132*, *FHL2*, and *TRIM59*) according to the correlation coefficient ranking (|*r*|≥0.8) and the *KLF14* gene by GEO data mining using a three-step feature selection algorithm AgeGuess (Gao et al., 2020), including a total of 34 CpGs (details in Supplementary Table S1). The PCR primers of five age-related DNAm biomarkers (Supplementary Table S2) were designed by PyroMark Assay Design Software 2.0 (Qiagen, Hilden, Germany).

A total of 240 unrelated healthy individuals were recruited from Han Chinese, who had settled in south China for at least three generations. Peripheral blood samples (2 ml) and accurate information (including age, gender, nationality) were collected from all participants of the CHS cohort. All volunteers had signed the informed consent forms (the underage children were signed by their guardians in accordance with Chinese laws and regulations), and the study was approved by the Biomedical Ethical Committee of Southern Medical University (No. 2021-015) following the standards of Declaration of Helsinki.

### Sample Preparation and BTA-pseq

#### DNA Extraction and Quantification

Genomic DNA was extracted from 200 μl peripheral blood by QIAamp Blood Mini Kit (Qiagen, Hilden, Germany) according to the manufacturer’s protocol. The extracted DNA samples were then quantified using Qubit^®^ 4.0 Fluorometer instrument (Thermo Fisher Scientific, Waltham, MA, United States) with Qubit^®^ dsDNA HS Assay Kit (Thermo Fisher Scientific, Waltham, MA, United States) according to the manufacturer’s instructions.

#### Bisulfite Conversion

The conversion of unmethylated cytosines to uracils in DNA samples was carried out with the EpiTect Fast DNA Bisulfite Kit (Qiagen, Hilden, Germany), following the manufacturer’s instruction. With the input of 300 ng DNA, the bisulfite DNA conversion was performed using a thermal cycler that comprised: two cycles of initial denaturation at 95°C for 5 min and incubation at 60°C for 10 min followed by a hold at 20°C for up to 20 h in the thermal cycler. The converted DNA was then eluted into 15 μl of the elution buffer (EB) obtained from the same kit, normalized to 20 ng/μl as the DNA template, and subsequently stored at −20°C until use.

#### Targeted Amplicon PCR

After bisulfite conversion, 100 ng of each converted DNA was submitted into a multiplex polymerase chain reaction (PCR) amplification with PyroMark PCR Kit (Qiagen, Hilden, Germany). Each multiplex reaction was performed in a final volume of 25 μl containing 12.5 μl of 2✕ PyroMark PCR Master Mix (providing a concentration of 1.5 mM MgCl_2_), 2.5 μl of 10✕ CoralLoad Concentrate, 9 μl of primer mix, and 1 μl of template DNA. The multiplex reaction was amplified under the following conditions: 1) initial PCR activation at 95°C for 15 min; 2) 45 cycles consisting of denaturation at 94°C for 30 s, annealing at 56°C for 30 s, and extension at 72°C for 30 s; and 3) final extension at 72°C for 10 min followed by a hold at 4°C. Negative control without DNA template was prepared in each PCR process.

#### Pyrosequencing

Following amplification, all PCR products were sequenced using PyroMark Gold Q24 Reagents (Qiagen, Hilden, Germany) in combination with PyroMark Q24 platform (Qiagen, Hilden, Germany) according to the manufacturer’s instructions. The generated pyrogram traces with sharp and distinct peaks were subsequently analyzed, and the methylation levels at different CpGs were calculated by the peak heights observed in PyroMark Q24 Advanced software v3.0.1 (Qiagen, Hilden, Germany). The missing methylation percentage values have been filled in with the median (Supplementary Table S3).

### Statistical Analysis

#### Spearman Correlation

The Spearman correlation coefficient (*r*) was calculated by IBM^®^ SPSS^®^ Statistics 26 (IBM Corporation, Armonk, NY, United States), SAS^®^ 9.4 software (SAS Institute Inc., Cary, NC, United States), and R (version 3.6.1). The *r* values are used to assess the strength and direction of the linear relationships between pairs of variables (predicted and chronological ages). According to Mukaka (2012), the *r* values followed the rule of thumb for interpreting size of a correlation coefficient: 1) 0.9≤*|r|*≤1.0, very high correlation; 2) 0.7≤*|r|*<0.9, high correlation; 3) 0.5≤*|r|*<0.7, moderate correlation; and 4) *|r|*≤0.5, low correlation. The AR-CpGs (*|r|*≥0.7) were selected to establish different ML models.

#### Dataset Information

**FIGURE 2:**
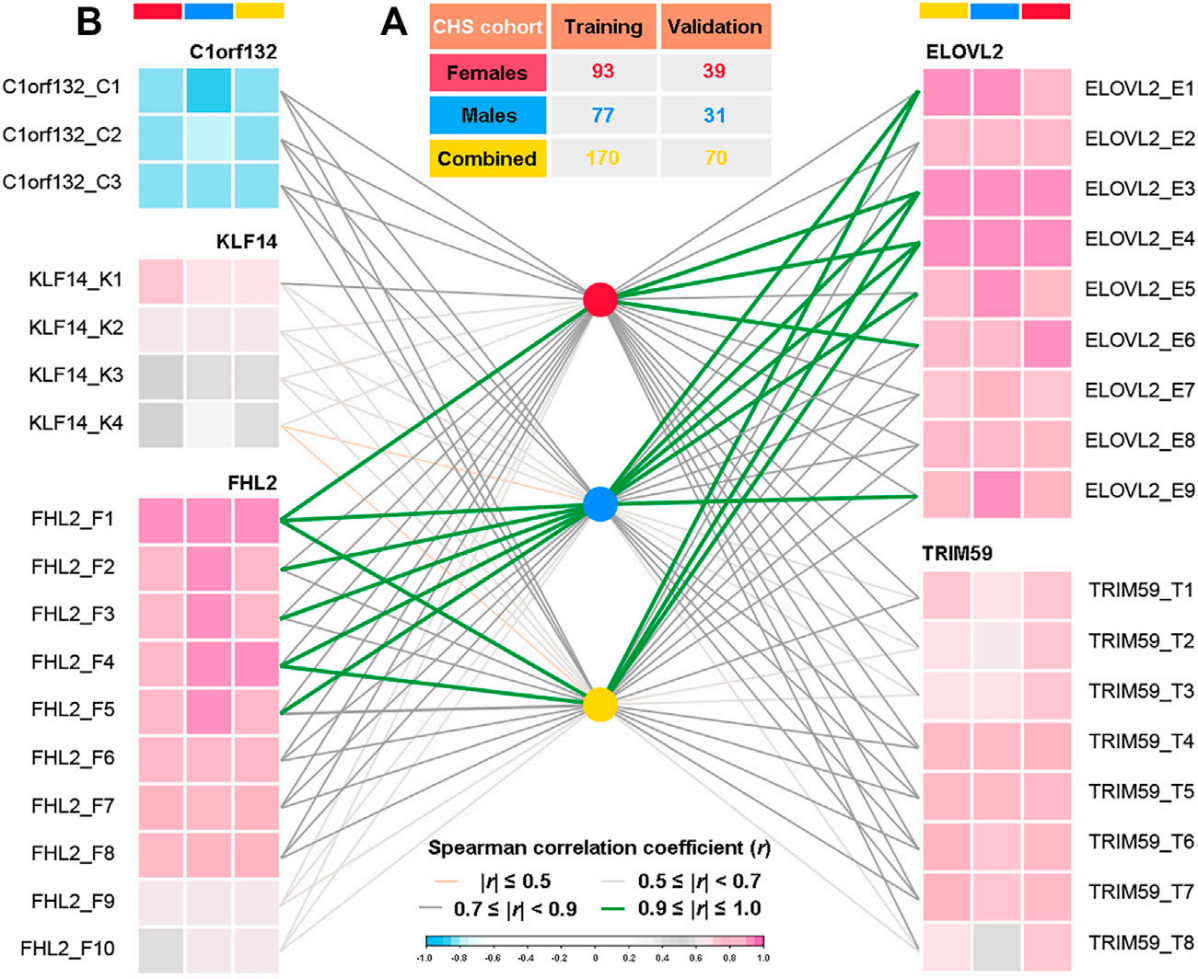
Spearman correlation analyses between DNA methylation levels of 34 CpGs located at five genes and chronological ages of three different datasets in the CHS cohort (*n* = 240, blood samples). **(A)** Detailed population sizes of different datasets (in the CHS cohort, randomly 70%/30% for Training and Validation sets, detailed information in Supplementary Table S4). **(B)** Spearman correlations between chronological ages and DNA methylation levels at each CpG in three different gender datasets (*r*, correlation coefficient; 0.9≤*|r|*≤1.0, very high correlation; 0.7≤*|r|*<0.9, high correlation; 0.5≤*|r|*<0.7, moderate correlation; *|r|*≤0.5, low correlation, details in Supplementary Table S5).

As shown in Figure 2A, the CHS cohort was randomly divided into a Training set (70%, *n* = 170, 93 females and 77 males) and a Validation set (30%, *n* = 70, 39 females and 31 males). The obtained methylation levels of Training set and the corresponding chronological ages were used for model training. Parameter tunning was performed by leave-one-out (*k*-fold) cross-validation, during which a set of samples (a fold) is removed from the dataset as the Validation set and the remaining samples were assigned as a Training set. In addition, for the evaluation of gender differences and aging effects, both Training and Validation sets were divided into three different gender datasets (female, male, and combined datasets, details in Figure 2A) and four age categories (1–20, 21–40, 41–60, and 61–81 years, details in Supplementary Table S4), respectively.

#### Model Performance Comparison

Model performance was compared in terms of *MAD* and *RMSE* values, which are calculated by IBM^®^ SPSS^®^ Statistics 26 and R (version 3.6.1). The *MAD* value is defined as the average distance between each data value and the mean, a way to describe variation in a dataset, while the *RMSE* value is widely used to compute the error distance between the estimated values. Both of them are the main metrics used to measure the quality of the regression output models. To measure the overall performance of each model, the *MAD* and *RMSE* values were calculated for the whole CHS cohort. Subsequently, to evaluate the generalization and the actual prediction performance of the final model, and to evaluate gender or aging effects, *MAD* values for different datasets needed to be analyzed.

### Machine Learning Model

#### Stepwise Regression Model

For multivariate linear regression analysis, the model selection procedure SR was performed using IBM^®^ SPSS^®^ Statistics 26 (IBM Corporation, Armonk, NY, United States) for model building together with 0.05 significance criteria for inclusion in the final model. Specifically, by excluding all previously selected variables with a *p*-value of 0.05 or greater until no variables can be eliminated nor new variables can be introduced in the regression equation, *stepwiselm* can create a linear model and automatically add to or trim the model, thus improving the selection of important variables in relatively small datasets (Núñez et al., 2011). Overall, the essence of these steps is to establish an “Optimal” MLR equation. The accuracy of age prediction with those tested CpGs was assessed by the goodness-of-fit (*R* ^*2*^), which is a parameter establishing the discrepancy between the observed values (chronological ages) and the expected values (predicted ages) under an applicable model, and generally used in regression to evaluate the performance of the model. Therefore, model equations with the greatest *R* ^*2*^ were selected as the candidate predictors based on the multivariant regression analysis.

#### Support Vector Regression Model

For SVR analysis, SVR model was carried out by R (*e1071* package). As reported, support vector machine (SVM) is a powerful technique for classification, regression, and outlier detection, and a correct choice of kernel parameters is crucial for a promising result. So, we constructed and refined regression models by following methods: 1) select support vector machines with radial (SVMr) function as kernel, 2) employ eps-regression and nu-regression for comparison, and 3) adjust the parameters “cost, gamma, and epsilon” for eps-regression and “cost and nu” for nu-regression. Eventually, two optimized SVR models with best-performing parameters were obtained.

#### Random Forest Regression Model

For random forest regression analysis, random forest exploiting classification trees were constructed based on Breiman’s random forest algorithm (on the basis of Breiman and Cutler’s original Fortran code) using *randomForest* R package. Random forests represent an effective tool in prediction, and RFR algorithm that based on decision trees plays an important role in selecting the “optimal” markers for model building. To reduce bias and operate effectively in regression, optimization of the RFR model was carried out by tuning the parameters *mtry* and *ntree*. *mtry* refers to the number of variables randomly sampled as candidates at each split, and *ntree* is defined as the number of trees to grow. By multiple rounds of optimization, a final *mtry* of 8 was chosen, the *ntree* was set at 500, and the optimal RFR model was established. The value (% Var explained) represents the overall explanatory rate for the variances of the response variables by the predictive variables. We used the value (%IncMSE, increase in mean squared error) to measure the importance of predictive variables, which means that by randomly assigning a value to each predictive variable, if the predictive variable is more important, the model prediction error will increase after its value is randomly replaced.

## Results

### AR-CpG Selection and Spearman Correlation

At first, a cohort of 7,084 individuals from 16 countries or populations related to DNAm-based age prediction studies was collected by bibliographic search to conduct meta-analyses (details in Supplementary Figure S1). Figure 1A presents the results of a meta-analysis of the detailed correlation coefficients for candidate age-associated genes in the meta cohort. The absolute values of correlation coefficients (*|r|*) for nine DNAm biomarkers ranged from 0.59 (ZNF423) to 0.89 (*ELOVL2*). There are eight of nine DNA biomarkers with *|r|*≥0.7 (Figure 1), and the *|r|* ranking of the candidate genes is visualized in Figure 1B. According to the self-defined threshold value (*|r|*≥0.8), four promising genes (*ELOVL2*, *C1orf132*, *FHL2*, and *TRIM59*) were selected for further validation in the CHS cohort. In addition, the *KLF14* gene that was screened by a three-step feature selection algorithm AgeGuess (Gao et al., 2020) was also selected. Supplementary Tables S1, S2 present the detailed 34 CpGs and PCR primers of five aforementioned DNAm biomarkers, respectively.

The detailed DNAm levels of 34 CpGs and the corresponding personal information (chronological ages and genders) in the CHS cohort are presented in Supplementary Table S3. In addition, according to gender stratification (Figure 2A and Supplementary Table S4), the Spearman correlation analyses were conducted between the DNAm levels and the chronological ages in three different datasets, which is visualized in Figure 2B (detailed results in Supplementary Table S5). Except for *C1orf132* where DNAm decreases with age, other genes have positive correlations with chronological ages. In total, we identified 25 AR-CpGs out of the 34 CpGs in the CHS cohort (29 AR-CpGs for female dataset, 24 AR-CpGs for male dataset), which are highly related (*|r|*≥0.7, *p* < 0.05) with the chronological ages of CHS. In addition, the *KLF14* has no apparent strong correlation with the chronological ages (all *r* < 0.7), except for KLF14_K1 in males (*r* = 0.7082). Meanwhile, three different AR-CpGs (ELOVL2_E3, ELOVL2_E4, and FHL2_F1) have high correlations with the chronological ages in all gender datasets of the CHS cohort. Detailed results of Spearman analyses are visualized in Supplementary Figures S2–S6 for *ELOVL2*, *C1orf132*, *FHL2*, *TRIM59*, and *KLF14*, respectively.

### Stepwise Regression Model

The AR-CpGs with *|r|*≥0.7 of different datasets were regarded as alternative stepwise variables. A stepwise variable selection was conducted to select the best possible combination of predictors from the candidate highly associated CpGs for the SR model, which guaranteed the explained variability without overfitting the data. Based on different gender datasets, we built three distinct SR equations and calculated corresponding statistics for female (*MAD* = 3.00 and *RMSE* = 4.07), male (*MAD* = 2.64 and *RMSE* = 3.45), and combined (*MAD* = 2.97 and *RMSE* = 3.89) datasets corresponding to the age prediction models (details in Table 1, all adjusted *R* ^*2*^ ≥ 0.93). There was no significant difference between females and males in the CHS cohort (*t* = 0.59, *p* = 0.61).

**TABLE 1:** Stepwise regression (SR) equations and system efficiencies in three different datasets of the CHS cohort (*n* = 240, blood samples).

Dataset	SR equation	*R* ^2^	Adjusted *R* ^2^	*RMSE*	*MAD*
Females	y = 35.518 + 0.679×F1−0.317×C1+0.319×T2−0.241×C2+0.438×E2+0.170×T4−0.202×F4+0.124×K1	0.94	0.93	4.07	3.00
Males	y = 21.347 + 0.488×E1−0.412×C1+0.360×F5+0.125×E7+0.320×E5	0.96	0.96	3.45	2.64
Combined	y = 24.260 + 0.348×F1−0.463×C1+0.188×E3+0.151×E1+0.088×T4+0.315×E2−0.260×F4+0.222×F2+0.054×E7+0.125×T5	0.95	0.94	3.89	2.97

R^2^, coefficient of determination/goodness-of-fit; Adjusted R^2^, adjusted coefficient of determination; RMSE, root mean square error; MAD, median absolute deviation.

Furthermore, we evaluated the prediction accuracy of the SR models in Training (*MAD* = 3.04, *n* = 170) and Validation (*MAD* = 2.80, *n* = 70) sets, respectively (Supplementary Table S6). The *MAD* values between Training and Validation sets had no significant difference (*t* = −1.06, *p* = 0.31). In total, the *MAD* values of different CHS datasets ranged from 2.14 (1–20 age category of Training set, *n* = 41) to 5.12 (61–81 age category of Validation set, *n* = 3). In addition, in the female dataset, the *MAD* values spanned from 2.25 (1–20 age category of Training set, *n* = 20) to 8.39 (61–81 age category of Validation set, *n* = 1). In the male dataset, the *MAD* values varied from 1.91 (1–20 age category of Validation set, *n* = 9) to 6.73 (61–81 age category of Validation set, *n* = 2). For different age categories, the lowest *MAD* value (1.91) was found at male validation dataset (1–20 age category, *n* = 9), while the highest *MAD* value (8.39) was identified at female validation dataset (61–81 age category, *n* = 1). The *MAD* values between females and males had no significant difference in both Training (*t* = 1.06, *p* = 0.35) and Validation (*t* = 0.25, *p* = 0.54) sets. Apparently, the *MAD* values rise with advancing ages (especially in the 61–81 age category), which indicated that the methylation-based SR model prediction accuracy decreases due to biological and physiological changes involved in the aging process, especially for the aged.

### Support Vector Regression Model

Here, we constructed SVR models with two different methods (eps- and nu-regression) using correspondingly AR-CpG loci (*|r|*≥0.7) of distinct gender groups.

#### SVR eps-Regression

As shown in Table 2, we found 163 support vectors in the CHS cohort with an *MAD* value of 2.22 (*RMSE* = 2.95). In addition, the *MAD* values were 2.09 and 2.12 for female (*n* = 132, *RMSE* = 2.84) and male (*n* = 108, *RMSE* = 2.93) datasets, respectively, with no significant difference (*t* = 0.51, *p* = 0.13). The best performance (with the lowest *MAD* value) of SVR eps-regression was obtained with the optimized parameters (cost = 1, gamma = 0.04, epsilon = 0.1). The detailed *MAD* values for Training and Validation sets are presented in Supplementary Table S7. The *MAD* values were 2.33 and 1.87 for Training and Validation sets, respectively, with no significant difference (*t* = 1.68, *p* = 0.12).

**TABLE 2:** Model settings and system efficiencies for three different datasets of the CHS cohort (*n* = 240, blood samples) in two SVR models.

SVR	Setting				Dataset	*n*	Number of support vectors	*RMSE*	*MAD*
	cost	gamma	epsilon	nu					
SVR-eps	1	0.04	0.1	–	Females	132	90	2.84	2.09
					Males	108	69	2.93	2.12
					Combined	240	163	2.95	2.22
SVR-nu	1	–	–	0.5	Females	132	105	2.82	1.92
					Males	108	79	2.90	2.00
					Combined	240	168	2.94	2.19

SVR-eps, support vector regression eps-regression; SVR-nu, support vector regression nu-regression; RMSE, root mean square error; MAD, median absolute deviation.

In different age categories, the *MAD* values ranged from 1.59 (1–20 age category of Validation set, *n* = 18) to 4.72 (61–81 age category of Training set, *n* = 12). In addition, in the female dataset, the *MAD* values spanned from 1.35 (1–20 age category of Validation set, *n* = 9) to 10.06 (61–81 age category of Training set, *n* = 4). In the male dataset, the *MAD* values varied from 1.53 (1–20 age category of Validation set, *n* = 9) to 5.09 (61–81 age category of Validation set, *n* = 2). The *MAD* values between females and males had no significant difference in both Training (*t* = 0.77, *p* = 0.07) and Validation (*t* = −0.38, *p* = 0.90) sets. Overall, except for the 61–81 age category, the *MAD* value for each dataset was no more than 2.44.

#### SVR nu-Regression

Besides, the SVR nu-regression model was also used to predict the chronological ages (Table 2). The *MAD* value of the CHS cohort was 2.19 (*RMSE* = 2.94), which was obtained at cost = 1 and nu = 0.5 (including 168 support vectors). In female and male datasets, the *MAD* values were 1.92 and 2.00 with the support vectors of 105 and 79, and the *RMSE* values were 2.82 and 2.90, respectively. However, there was no significant difference between females and males in the CHS cohort (*t* = 0.52, *p* = 0.09). The detailed *MAD* values of Training and Validation sets are presented in Supplementary Table S7. The *MAD* values were 2.33 and 1.84 for Training and Validation sets with no significant difference (*t* = 1.78, *p* = 0.10), respectively.

In different age categories, the *MAD* values ranged from 1.56 (1–20 age category of Validation set, *n* = 18) to 4.73 (61–81 age category of Training set, *n* = 12). In the female dataset, the *MAD* values spanned from 1.08 (1–20 age category of Validation set, *n* = 9) to 10.54 (61–81 age category of Training set, *n* = 4). In the male dataset, the *MAD* values varied from 1.27 (1–20 age category of Validation set, *n* = 9) to 5.18 (61–81 age category of Validation set, *n* = 2). The *MAD* values between females and males had no significant difference in both Training (*t* = 0.75, *p* = 0.07) and Validation (*t* = −0.27, *p* = 0.68) sets. The *MAD* value for each dataset was no more than 2.41 except for the 61–81 age category.

Compared with SVR-eps, the prediction capacity of the SVR-nu model was more excellent with lower *MAD* value for each dataset, while the model stability for both of them has larger fluctuations at the 61–81 age category (*MAD* values ranging from 3.42 to 10.54, details in Supplementary Table S7).

### Random Forest Regression Model

**FIGURE 3:**
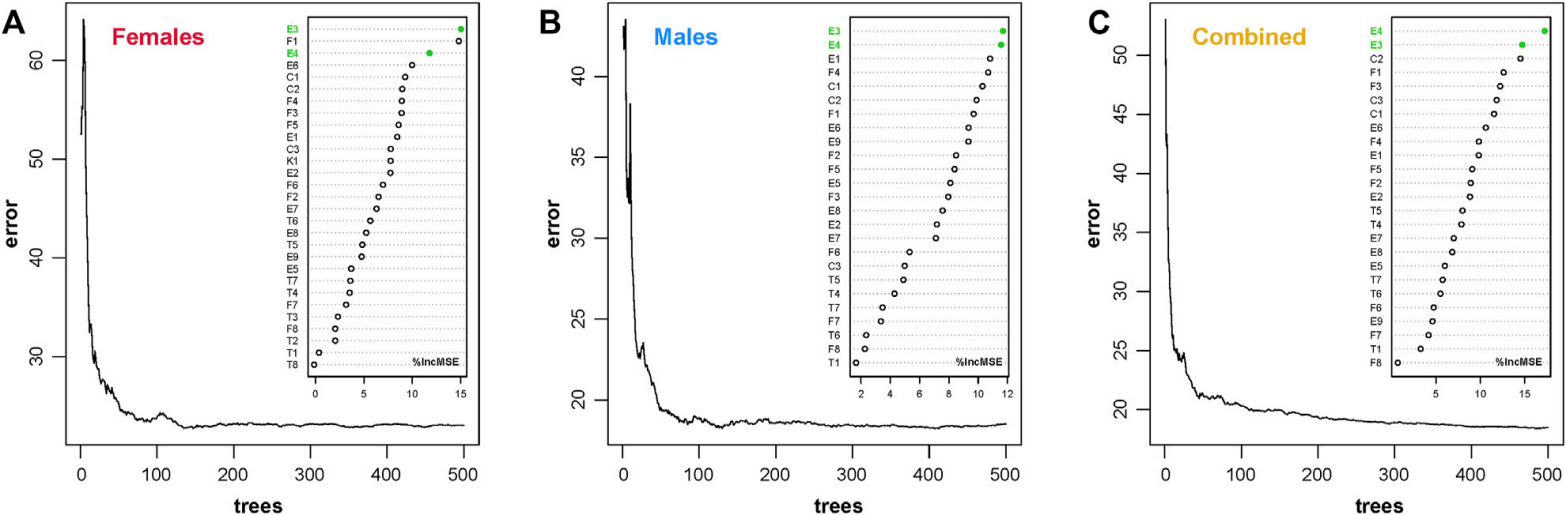
Validation of feature selection (*ntree* = 500) and AR-CpG importance ranking in three different gender datasets of the CHS cohort (*n* = 240, blood samples). **(A)** Female dataset (*n* = 132). **(B)** Male dataset (*n* = 108). **(C)** Combined dataset (*n* = 240). (***ntree***, number of trees to grow, which should not be set to too small a number, to ensure that every input row gets predicted at least a few times; **%IncMSE**, increase in mean squared error.)

Furthermore, the DNAm profiles of 240 CHS samples were learned by the RFR algorithm. For the *ntree* feature selection, we set six different threshold values (100, 300, 500, 1,000, 5,000, and 10,000) to find the robust limit with lower error rate (details in Supplementary Figure S7). In fact, the error rates tended to be stable when the *ntree* was more than 300. However, we set an *ntree* border at 500 to obtain more reliable results without regard to the hashrate for practice case handling. In addition, the feature selection (*ntree* = 500) was validated in different gender datasets, which indicated that the relatively lower and stable error rates are obtained with *ntree* of 500 (Figure 3). The E3 and E4 AR-CpG markers of *ELOVL2* genes (*r* > 0.9 in different gender datasets, details in Supplementary Table S5) ranked the top three positions in different gender datasets, which demonstrated that these biomarkers are the important predictive variables in the CHS cohort. According to different numbers of AR-CpGs for distinct gender datasets, the *mtry* values were set up at 9, 8, and 8 for female, male, and combined datasets, respectively.

With the feature selection and parameter setting as described above, the RFR model could explain 93.21% of the total variances (90.62% for females and 93.88% for males) in the CHS cohort (Table 3). The *MAD* values were 1.29 (*RMSE* = 1.77), 1.45 (*RMSE* = 1.95), and 1.32 (*RMSE* = 1.77) for combined, female, and male datasets, respectively. There was no significant difference between females and males in the CHS cohort (*t* = 0.98, *p* = 0.05). As shown in Supplementary Table S8, the *MAD* values of Training and Validation sets were 1.37 and 1.10, with no significant difference (*t* = 1.97, *p* = 0.07).

**TABLE 3:** Detailed feature selection and model efficiency information of random forest regression (RFR) models in three different gender datasets of the CHS cohort.

ML model	Dataset	n	*ntree*	*mtry*	% Var explained	*RMSE*	*MAD*
RFR	Females	132	500	9	90.62	1.95	1.45
	Males	108	500	8	93.88	1.77	1.32
	Combined	240	500	8	93.21	1.77	1.29
RFR (1–60)	Females	127	500	9	91.35	1.67	1.29
	Males	98	500	8	92.92	1.60	1.20
	Combined	225	500	8	93.13	1.54	1.15

ntree, number of trees to grow, which should not be set to too small a number, to ensure that every input row gets predicted at least a few times; mtry, number of variables randomly sampled as candidates at each split; % Var explained, the overall explanatory rate for the variances of the response variables by the predictive variables; RMSE, root mean square error; MAD, median absolute deviation.

In different age categories, the *MAD* values ranged from 0.45 (1–20 age category of Validation set, *n* = 18) to 3.39 (61–81 age category of Validation set, *n* = 3). In the female dataset, the *MAD* values spanned from 0.59 (1–20 age category of Validation set, *n* = 9) to 4.47 (61–81 age category of Training set, *n* = 4). In the male dataset, the *MAD* values varied from 0.75 (1–20 age category of Validation set, *n* = 9) to 2.21 (61–81 age category of Validation set, *n* = 8). The *MAD* values between females and males had no significant difference in both Training (*t* = 0.90, *p* = 0.13) and Validation (*t* = 0.39, *p* = 0.23) sets. The detailed *MAD* values for each dataset are presented in Supplementary Table S8, and except for the 61–81 age category, the *MAD* values were less than 1.80.

### Model Performance Comparison

**FIGURE 4:**
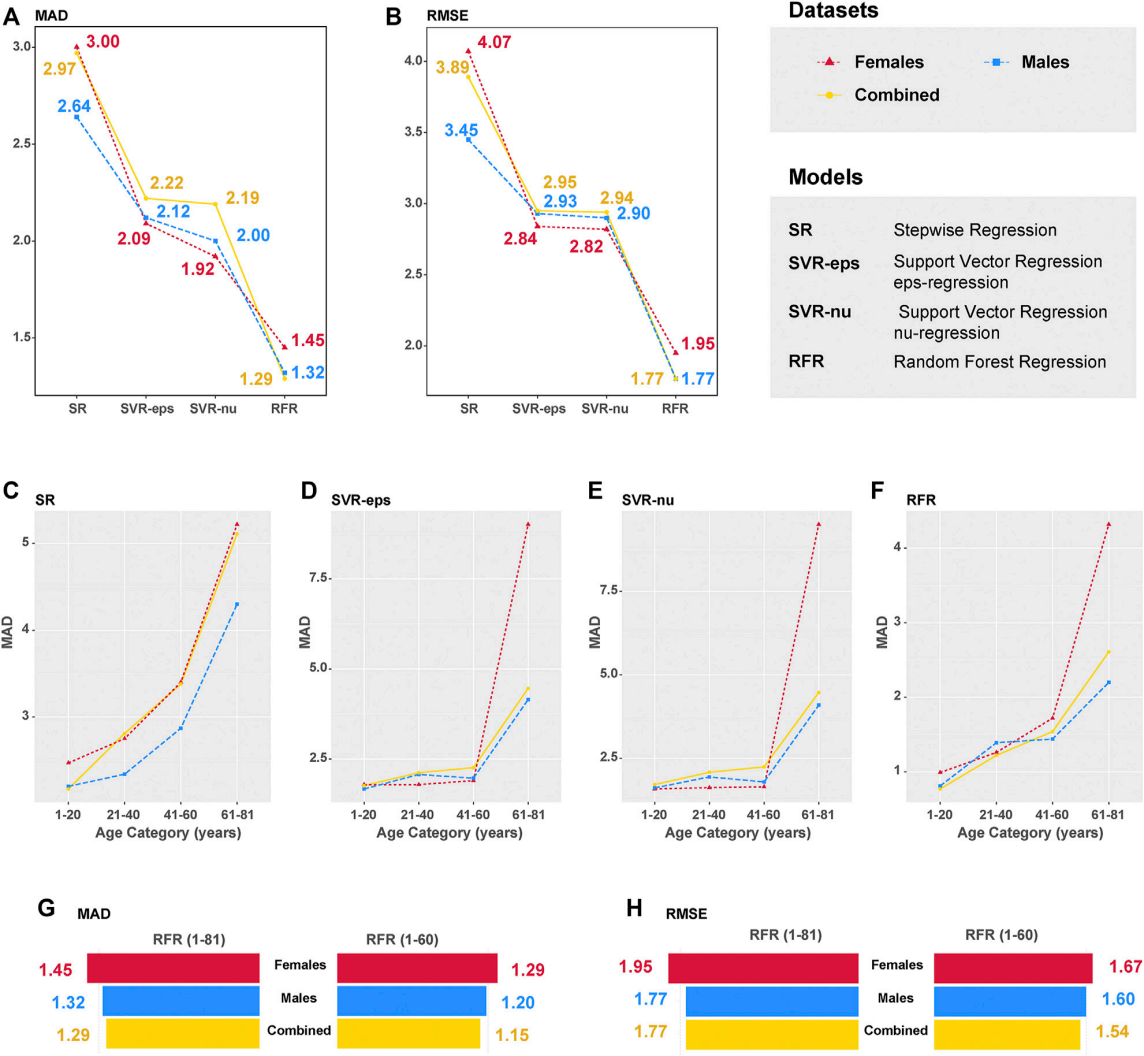
Median absolute deviation (*MAD*) and root mean square error (*RMSE*) values of different machine learning (ML) models. **(A)** *MAD* value comparison of four different models in the CHS cohort (*n* = 240, blood samples). **(B)** *RMSE* value comparison of four different models in the CHS cohort (*n* = 240, blood samples). **(C)** *MAD* values in different age categories of the SR model. **(D)** *MAD* values in different age categories of the SVR-eps model. **(E)** *MAD* values in different age categories of the SVR-nu model. **(F)** *MAD* values in different age categories of the RFR model. **(G)** *MAD* value comparison between RFR (1–81) and RFR (1–60) models. **(H)** *RMSE* value comparison between RFR (1–81) and RFR (1–60) models.

Based on aforementioned ML algorithms, four different ML models have been established after multiple rounds of optimization, and the model efficiencies have been evaluated (details in Table 4). All *R* ^*2*^ values were above 0.95, and the *R* ^*2*^ value reached to 0.99 in the RFR model. The *MAD* values of the CHS cohort were 2.97 (*RMSE* = 3.89), 2.22 (*RMSE* = 2.95), 2.19 (*RMSE* = 2.94), and 1.29 (*RMSE* = 1.77) for SR, SVR-eps, SVR-nu, and RFR models, which are also visualized in Figures 4A,B. In the female dataset, the *MAD* values were 3.00 (*RMSE* = 4.07), 2.09 (*RMSE* = 2.84), 1.92 (*RMSE* = 2.82), and 1.45 (*RMSE* = 1.95) for SR, SVR-eps, SVR-nu, and RFR models, respectively. In the male dataset, the *MAD* values were 2.64 (*RMSE* = 3.45), 2.12 (*RMSE* = 2.93), 2.00 (*RMSE* = 2.90), and 1.32 (*RMSE* = 1.77) for SR, SVR-eps, SVR-nu, and RFR models, respectively. It demonstrated that no matter in female or male datasets, the RFR model had the highest predictive accuracy with an *MAD* value of 1.29.

**TABLE 4:** System efficiency comparisons of different machine learning (ML) models.

ML model	*R* ^2^	*RMSE*	*MAD*
SR	0.95	3.89	2.97
SVR-eps	0.97	2.95	2.22
SVR-nu	0.97	2.94	2.19
RFR	0.99	1.77	1.29
RFR (1–60)	0.99	1.54	1.15

R^2^, coefficient of determination/goodness-of-fit; RMSE, root mean square error; MAD, median absolute deviation; SR, stepwise regression; SVR-eps, support vector regression eps-regression; SVR-nu, support vector regression nu-regression; RFR, random forest regression in the CHS cohort; RFR (1–60), random forest regression at the 1–60 age categories of the CHS cohort.

In four different ML models of the CHS cohort, we definitely observed that the *MAD* values increased with the chronological ages, especially in the 61–81 age category with a rapid increase (Figures 4C–F). In addition, to obtain more precise prediction accuracy, we evaluated the best-performing RFR model in the age categories of 1–60 (excluding the 61–81 age category). As presented in Supplementary Figure S8, the *ntree* feature (*ntree* = 500) was further validated in different gender datasets, and the E3 and E4 CpGs of *ELOVL2* were also the most important predictive variables in the RFR model (1–60 age categories). The *MAD* value of all 225 CHS samples reduced to 1.15 (*RMSE* = 1.54), and the *MAD* values were 1.21 and 1.01 for Training (*n* = 158) and Validation (*n* = 67) sets, respectively (Supplementary Table S9). In Table 4 and Figures 4G,H, the *MAD* values of the RFR (1–60) model were 1.29 in females (*RMSE* = 1.67) and 1.20 in males (*RMSE* = 1.60). Compared with the RFR model for the 1–81 age categories, both the *MAD* and *RMSE* values of RFR (1–60) have decreased, and the *MAD* values were especially less than 1.00 in the 1–20 age category (Supplementary Table S9), which demonstrated that the RFR (1–60) model is more suitable for the age precise prediction of youngsters. Additionally, the relationships between predicted ages and chronological ages in different ML models were conducted (Supplementary Figure S9), and the *R* ^*2*^ values of all different ML models were more than 0.94.

## Discussion

Forensic community has long been seeking for a molecular marker to facilitate age prediction from biological traces at crime scenes. The DNAm biomarkers served as the most promising information source for chronological age estimation, even though the aging process was impacted by both inherited genetic and environmental factors (Li et al., 2018; Morrow et al., 2020; Ryan et al., 2020; Mukherjee et al., 2021). Most of the existing studies selected their DNAm biomarkers based on these biomarkers’ biological relevance to the aging process (Zubakov et al., 2016), statistically correlations with the chronological ages (Shadrina et al., 2018), or feature selection algorithms (Gao et al., 2020). In this study, the correlation coefficient ranking of nine candidate DNAm biomarkers was obtained from a cohort of 7,084 individuals using meta-analysis. Among them, we selected four top-ranking genes (*ELOVL2*, *TRIM59*, *FHL2*, and *C1orf132*) and *KLF14* chosen by a three-step feature selection algorithm AgeGuess to generate the DNAm profiles of the CHS cohort by BTA-pseq technology.

Correlation of DNAm status in five abovementioned genes with chronological age has been very well documented in different tissues and cell types (Zubakov et al., 2016; Cho et al., 2017; Jung et al., 2019; Dias et al., 2020; Anaya et al., 2021; Pfeifer et al., 2021; Woźniak et al., 2021). Our Spearman correlation analysis detected different strongly related CpG (*|r|*≥0.9) numbers in male (10 AR-CpGs) and female (4 AR-CpGs) datasets, mainly in *ELOVL2* and *FHL2*. However, the *MAD* values had no significant difference between female and male datasets in different SR (*t* = 0.59, *p* = 0.61), SVR-eps (*t* = 0.51, *p* = 0.13), SVR-nu (*t* = 0.52, *p* = 0.09), and RFR (*t* = 0.98, *p* = 0.05) models. The results indicated that the effect of gender on age prediction has not been detected in the present study (all *p* > 0.05), which was in concordant with Koch and Wagner (2011). In contrast, some studies presented that DNAm in men changes 4% faster than that in women (Hannum et al., 2013) and the predicted age was higher in men than in women (Weidner et al., 2014; Zbieć-Piekarska et al., 2015b). The gender effect on age estimation is inconclusive; however, it is conclusive that there is no gender effect in our ML models at least.

The chosen methylomic biomarker *KLF14* has strongly age-associated relationships in Caucasians and Hispanics (Gao et al., 2020), but the age correlations were not apparent in the CHS cohort, Koreans, and Polish (Supplementary Table S10). In addition, we observed high *r* value of 0.798 (F7 of *FHL2*) in the CHS cohort, but the corresponding *r* value is 0.42 in Polish. In different East Asian populations, the *r* values were 0.67 and 0.87 at T8 of *TRIM59* in CHS and Koreans, respectively. Our results demonstrated that different populations have distinct methylation status under the same conditions, for both intercontinental and regional populations (termed as population-specific), which indicated that it is urgently necessary to determine the population-specific AR-CpGs available for practical application regionally.

This study further established four different ML models for chronological age prediction in the CHS cohort. Our results obtained from both Training and Validation sets are concordant in four different ML models (all *p* > 0.05), and the *MAD* values were less than 3.0 years (Table 4), which indicated that all ML models are robust in the CHS cohort. Based on the same five age-related genes, Zbieć-Piekarska et al. constructed the SR model in Polish with the *MAD* values of 3.4 and 3.9 in Training and Validation sets, respectively (Zbieć-Piekarska et al., 2015b). Another SR model exhibited an *MAD* value of 4.18 in 100 Korean blood samples (Cho et al., 2017). Jung et al. used multiplex methylation SNaPshot assay to establish the SR model using 150 Korean blood samples with the *MAD* values of 3.174 and 3. 478 in Training and Validation sets, respectively (Jung et al., 2019). Compared to the aforementioned SR models, the SR model of the CHS cohort showed higher prediction accuracy (*MAD* = 3.04 in Training set and *MAD* = 2.80 in Validation set). In addition, the *MAD* values of two optimized SVR models were 2.22 and 2.19 for SVR-eps and SVR-nu models (Table 2, Table 4), which were better than the SR model in the CHS cohort. Additionally, the RFR model with an *MAD* value of 1.29 was the best-performing ML model in the CHS cohort, which was confirmed at both Training (*MAD* = 1.45) and Validation (*MAD* = 1.32) sets without significant difference. Under the same condition, different ML algorithms have apparent influences on age prediction model accuracy.

In our data, we also found that the age prediction accuracy decreases with chronological age in different ML models (Figures 4C–F). As DNAm is a dynamic modification process, age-associated changes in DNAm have been well documented, and a previous study has identified that DNAm tends to increase with age on some CpG islands (Field et al., 2018). Moreover, the *MAD* values are affected by small sample size (only 15 individuals in the 61–81 age category of the CHS cohort), resulting in some biases for chronological age prediction. Thus, the absolute differences between predicted and chronological ages are larger in the categories of older people, which are also confirmed by previous studies (Zbieć-Piekarska, et al., 2015b; Hamano et al., 2016; Cho et al., 2017; Dias et al., 2020). Notably, the *MAD* value of the RFR model reduced to 1.15 years in the age range of 1–60. In the meta cohort, the *MAD* values ranged from 2.53 to 5.07 years. As far as we know, it is the best chronological age prediction model in Han Chinese.

In fact, the DNAm status reflects biological age rather than chronological age. However, DNAm estimated age can be considered as an “epigenetic clock,” which in many cases runs parallel with chronological age (Horvath, 2013; Marioni et al., 2015). The epigenetic clock of CHS can be established by four age-related genes and different ML algorithms. From our perspectives, finding more population-specific and age-associated genes, expanding larger sample sizes (Figures 4G,H), and optimizing ML algorithms will contribute to generating more precise epigenetic clocks for diverse human populations.

## Conclusion

In the present study, we conducted that 1) a candidate set of nine DNAm biomarkers was collected by meta-analysis with a number of 7,084 individuals; 2) the DNAm profiles of five promising genes were generated using BTA-pseq in the CHS cohort; and 3) four different ML models based on age-related CpGs (*|r|*≥0.7) were established and optimized in different datasets. In addition, we concluded that 1) gender effect has little influence on age prediction; 2) methylation levels at different CpGs exhibit population specificity; and 3) the age prediction accuracy decreases with chronological age. Eventually, an optimized RFR ML model with an *MAD* value of 1.15 has been established (*ntree* = 500 and *mtry* = 8) at the 1–60 age categories of CHS using whole blood DNAm data generated by BTA-pseq.

## Data Availability

The original contributions presented in the study are included in the article/Supplementary Material, further inquiries can be directed to the corresponding authors.
