# Supplementary material for: Chronological Age Prediction: Developmental Evaluation of DNA Methylation-Based Machine Learning Models
Source: Front Bioeng Biotechnol. 2022 Jan 24;9:819991. doi: 10.3389/fbioe.2021.819991 (PMC8819006; doi:10.3389/fbioe.2021.819991)
Supplement: Supplementary file 1 [file DataSheet1.pdf]

Supplementary Figure S1

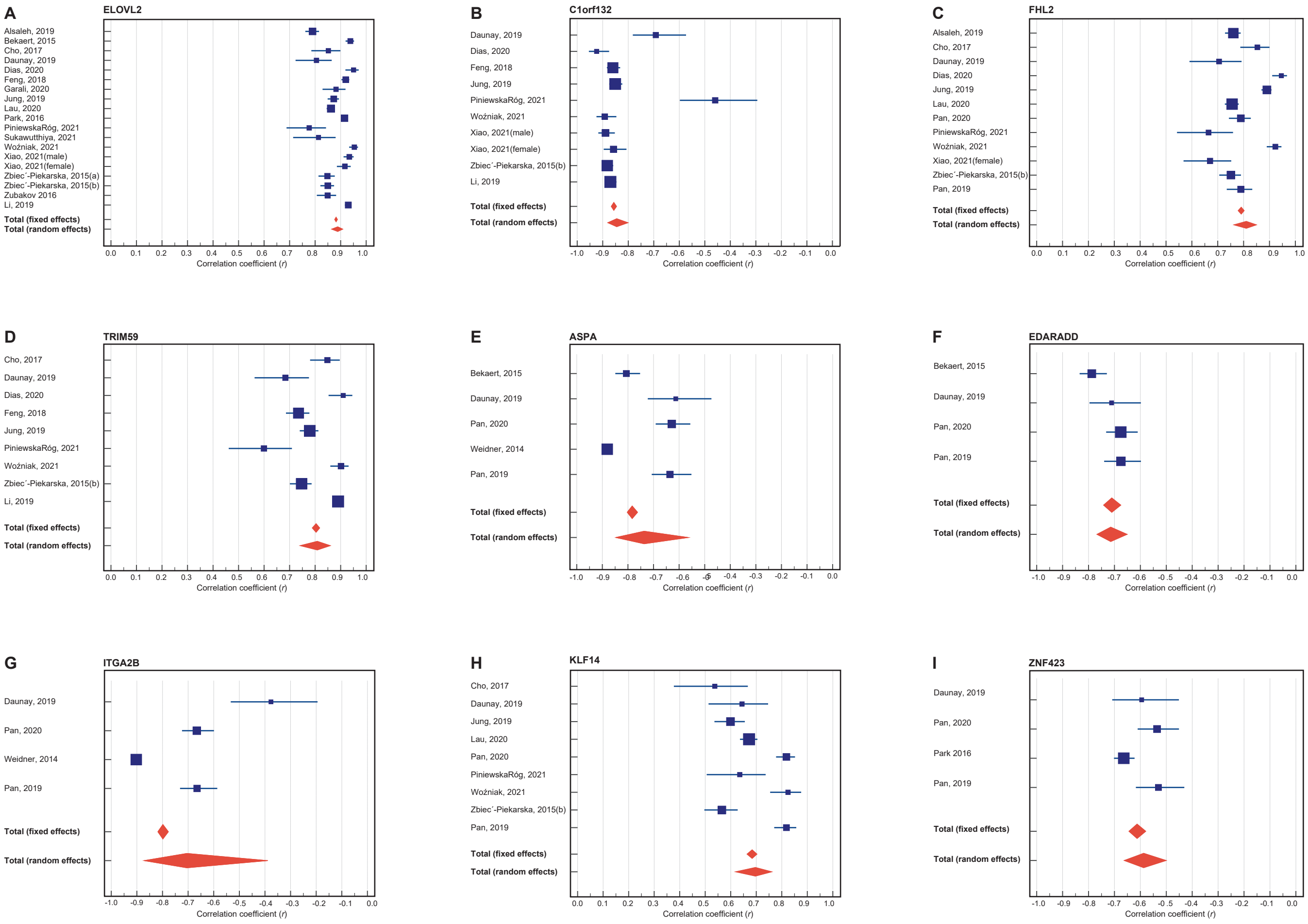

Supplementary Figure S1 Detailed correlation coefficient (*r*) meta-analysis forest plots of nine candidate DNA methylation biomarkers in meta cohort of 7084 individuals.
A. ELOVL2 (n = 5847, *r* = 0.89); **B.** C1orf132 (n = 2419, *r* = 0.85); **C.** FHL2 (n = 3768, *r* = 0.81); **D.** TRIM59 (n = 2206, *r* = 0.81); **E.** ASPA (n = 1421, *r* = 0.74); **F.** EDARADD (n = 846, *r* = 0.71); **G.** ITGA2B (n = 1215, *r* = 0.71); **H.** KLF14 (n = 2812, *r* = 0.70); **I.** ZNF423 (n = 1405, *r* = 0.59).

Supplementary Figure S2

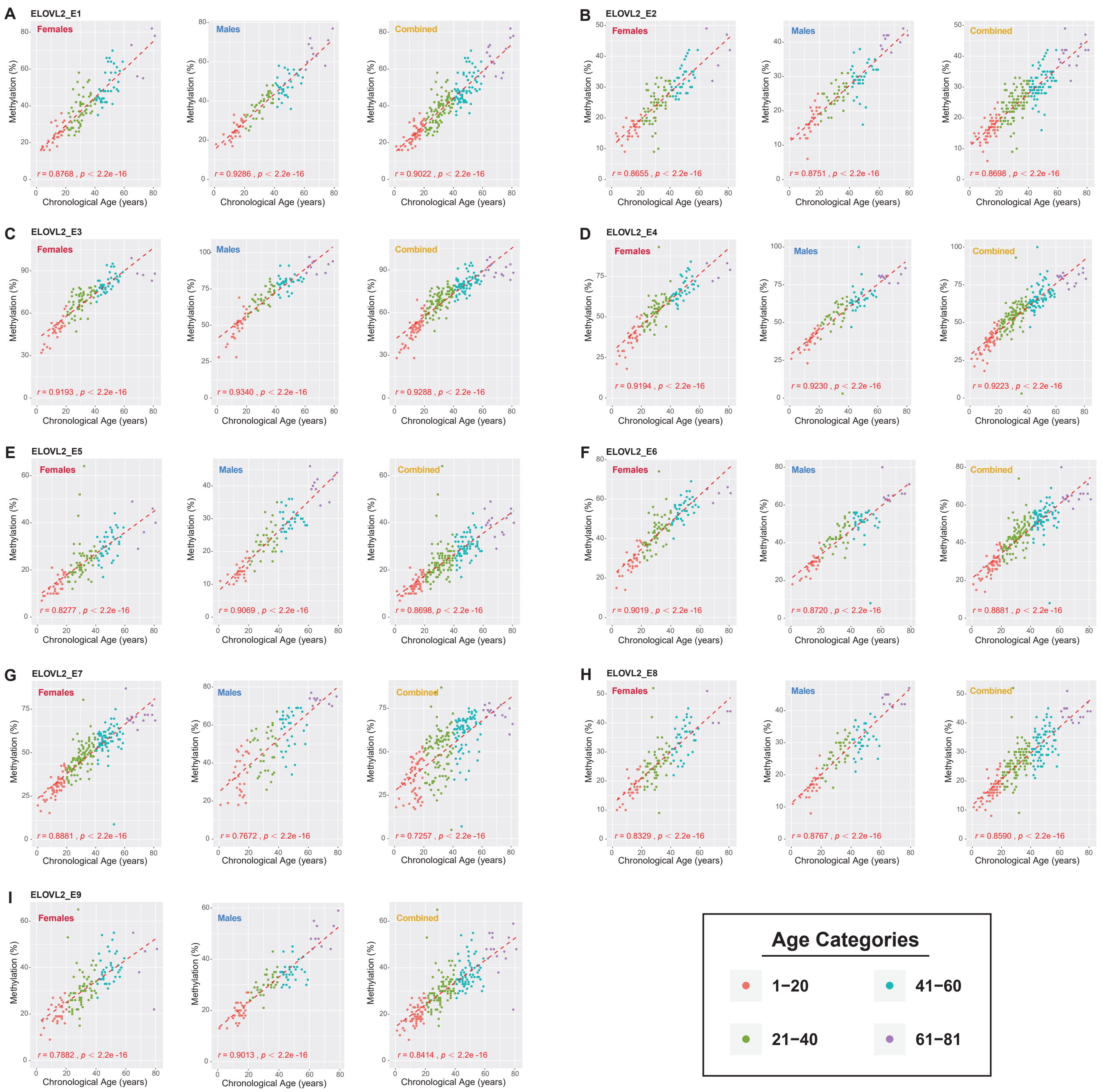

Supplementary Figure S2 Spearman correlation analyses between different CpG methylation levels of ELOVL2 and the chronological ages of different gender datasets in CHS cohort (n = 240, blood samples).

A. ELOVL2_E1; **B.** ELOVL2_E2; **C.** ELOVL2_E3; **D.** ELOVL2_E4; **E.** ELOVL2_E5;
F. ELOVL2_E6; **G.** ELOVL2_E7; **H.** ELOVL2_E8; **I.** ELOVL2_E9;
Detailed CpG information in Supplementary Table S1.

Supplementary Figure S3

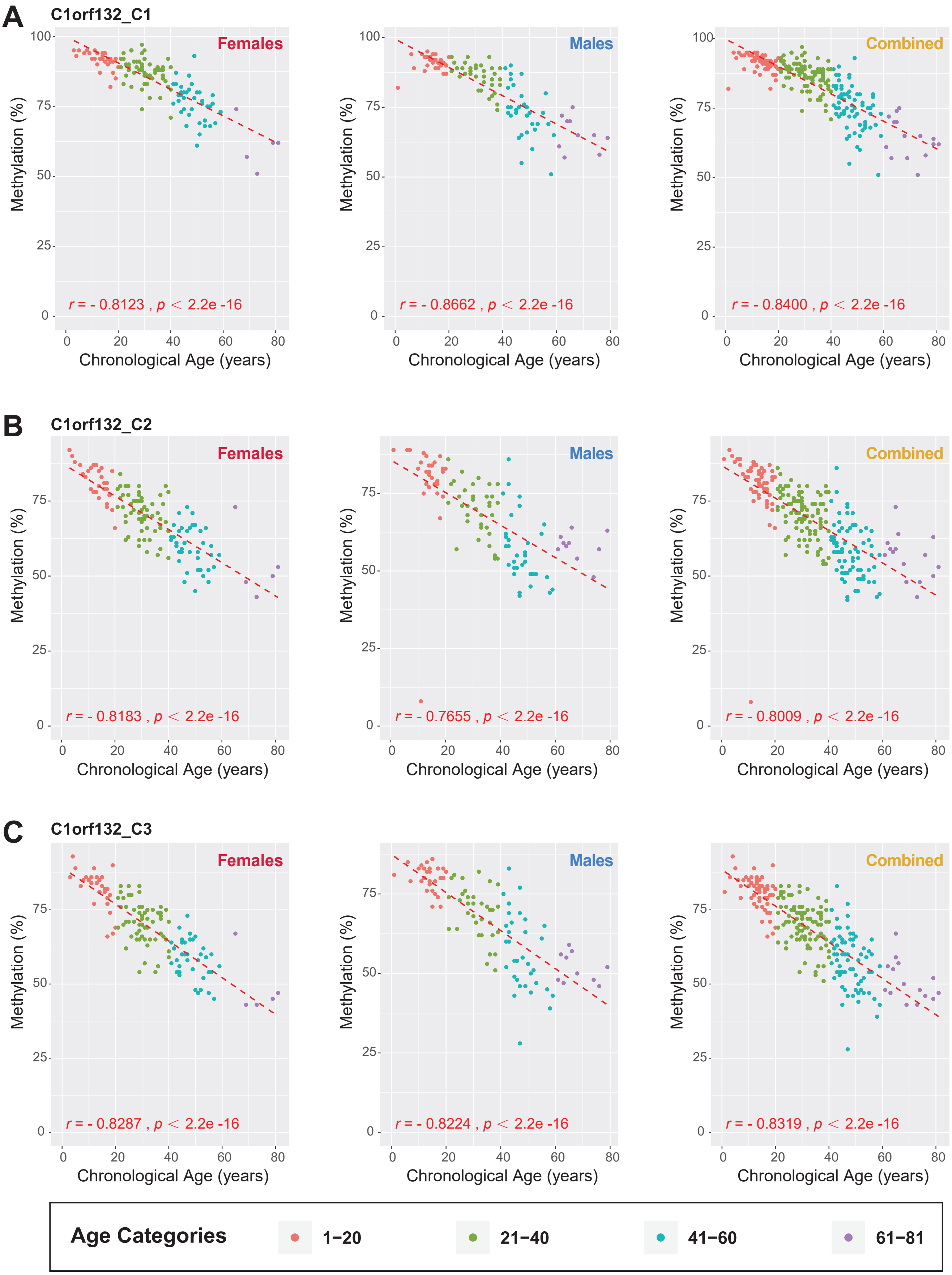

Supplementary Figure S3 Spearman correlation analyses between different CpG methylation levels of C1orf132 and the chronological ages of different gender datasets in CHS cohort (n = 240, blood samples).

A. C1orf132_C1; **B.** C1orf132_C2; **C.** C1orf132_C3; Detailed CpG information in Supplementary Table S1.

Supplementary Figure S4

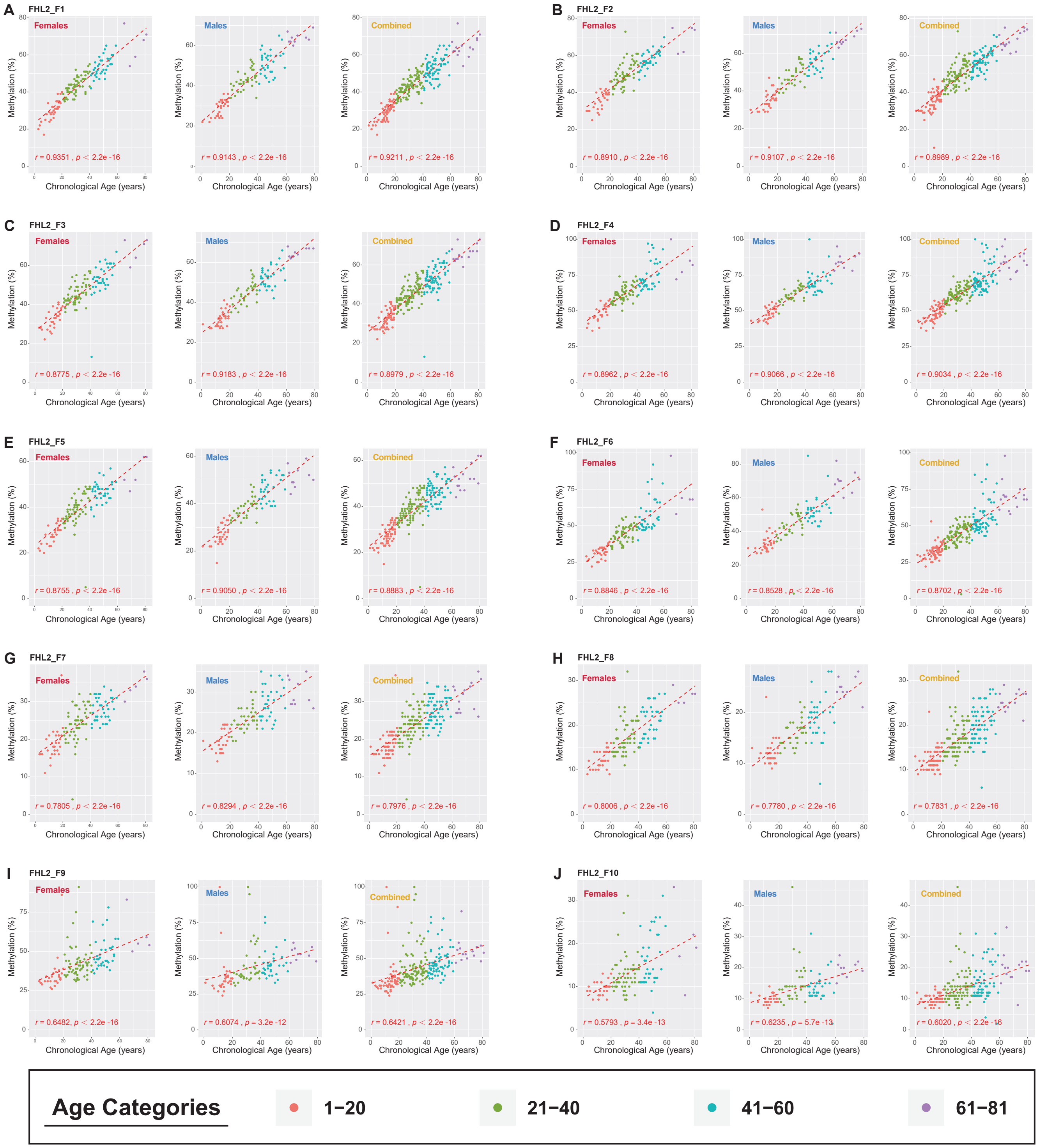

Supplementary Figure S4 Spearman correlation analyses between different CpG methylation levels of FHL2 and the chronological ages of different gender datasets in CHS cohort (n = 240, blood samples).

A. FHL2_F1; **B.** FHL2_F2; **C.** FHL2_F3; **D.** FHL2_F4; **E.** FHL2_F5;
F. FHL2_F6; **G.** FHL2_F7; **H.** FHL2_F8; **I.** FHL2_F9; **J.** FHL2_F10;
Detailed CpG information in Supplementary Table S1.

Supplementary Figure S5

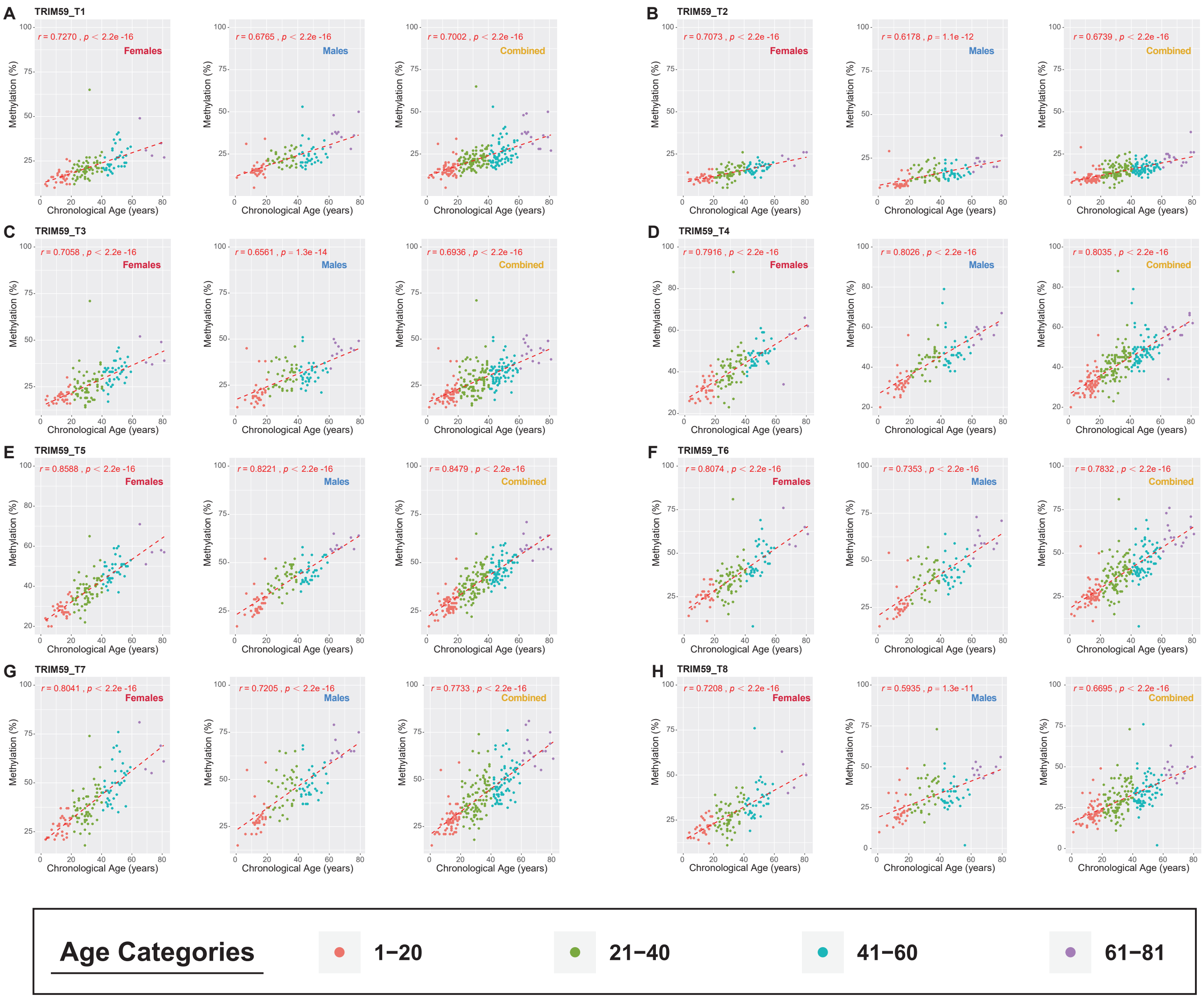

Supplementary Figure S5 Spearman correlation analyses between different CpG methylation levels of TRIM59 and the chronological ages of different gender datasets in CHS cohort (n = 240, blood samples).

A. TRIM59_T1; **B.** TRIM59_T2; **C.** TRIM59_T3; **D.** TRIM59_T4;
E. TRIM59_T5; **F.** TRIM59_T6; **G.** TRIM59_T7; **H.** TRIM59_T8;
Detailed CpG information in Supplementary Table S1.

Supplementary Figure S6

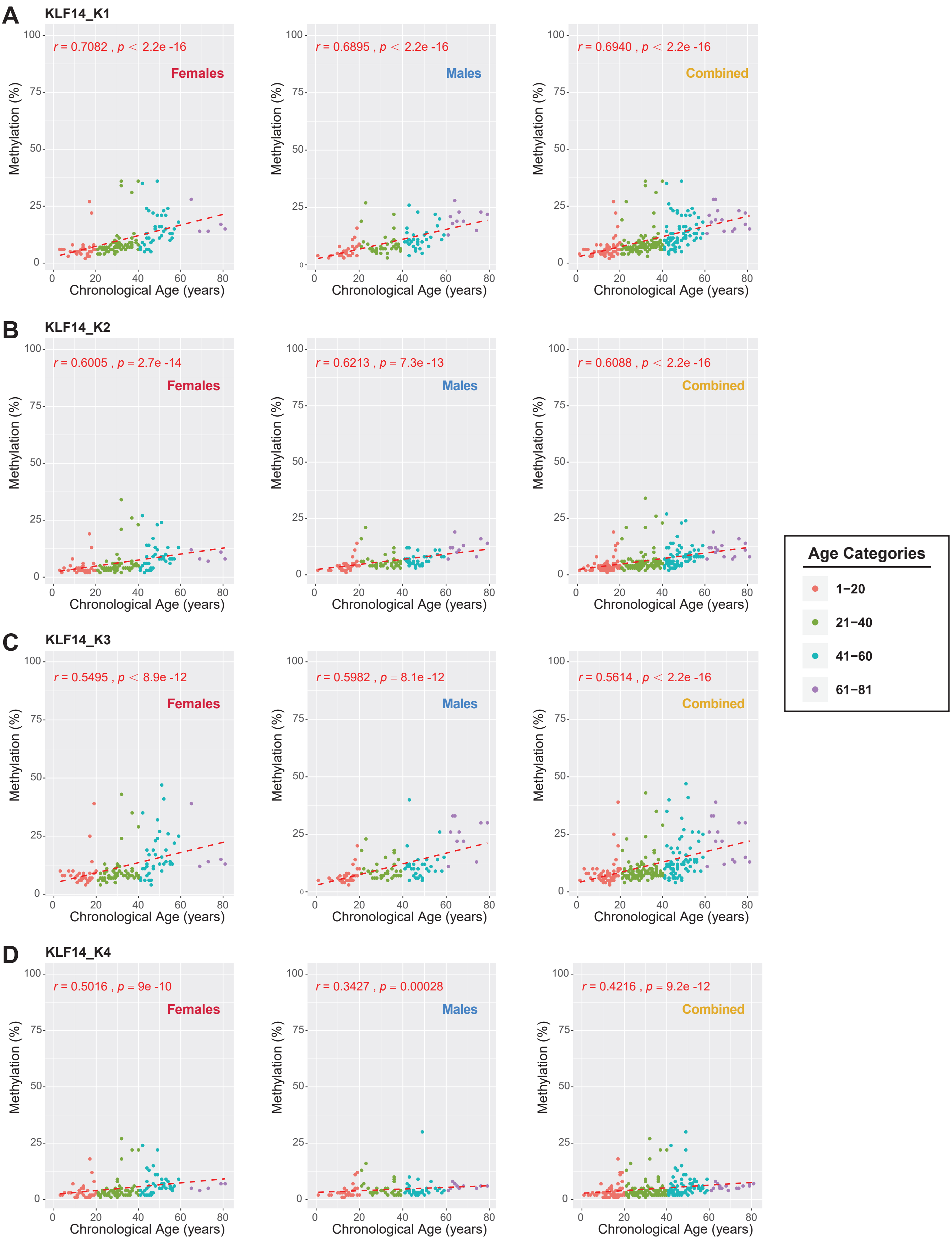

Supplementary Figure S6 Spearman correlation analyses between different CpG methylation levels of KLF14 and the chronological ages of different gender datasets in CHS cohort (n = 240, blood samples).

A. KLF14_K1; **B.** KLF14_K2; **C.** KLF14_K3; **D.** KLF14_K4; Detailed CpG information in Supplementary Table S1.

Supplementary Figure S7

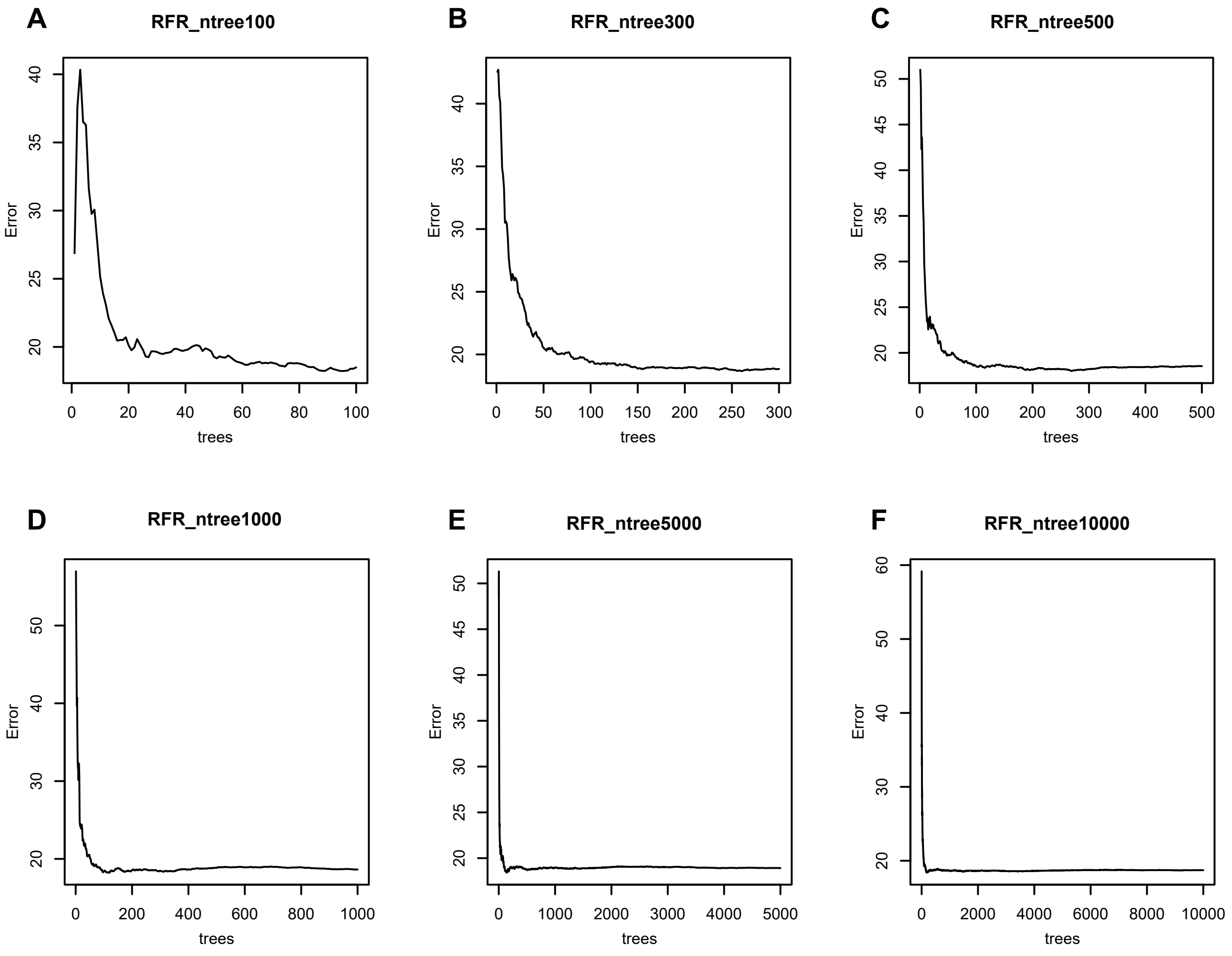

Supplementary Figure S7 Error rates of random forest regression (RFR) model at six different *ntree* features (100-10000) in CHS cohort (n = 240, blood samples).

A. *ntree* = 100; **B**. *ntree* = 300; **C**. *ntree* = 500; **D**. *ntree* = 1000; **E**. *ntree* = 5000; **F**. *ntree* = 10000.

(***ntree***, number of trees to grow, which should not be set to too small a number, to ensure that every input row gets predicted at least a few times.)

Supplementary Figure S8

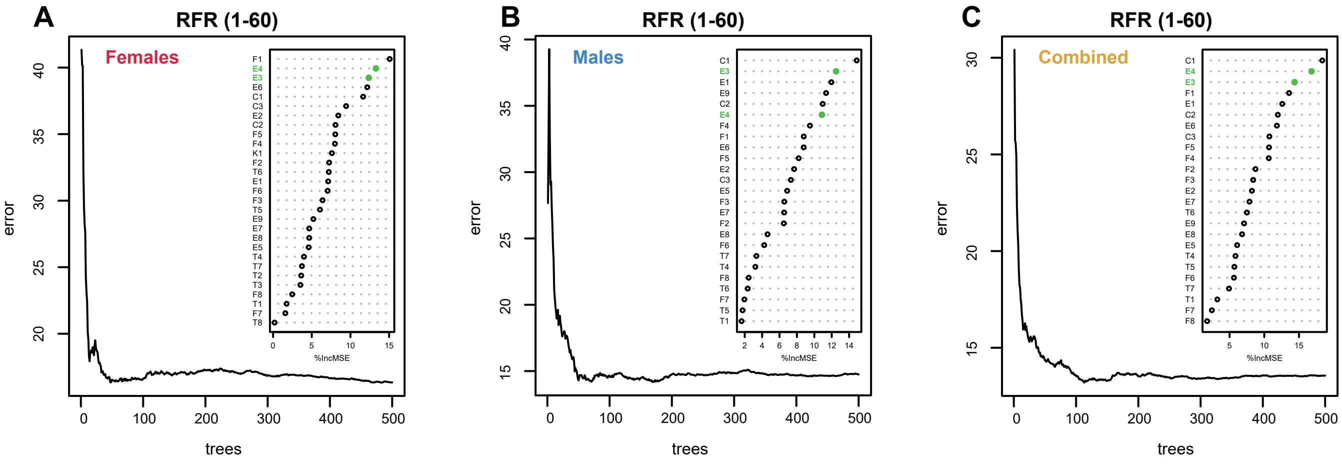

Supplementary Figure S8 Error rates (*ntree* = 500) and AR-CpG importance ranking of random forest regression (RFR) model at three different gender datasets of 1-60 age categories (n = 225, blood samples).

A. Female dataset of 1-60 age categories (n = 127); **B.** Male dataset of 1-60 age categories (n = 98); **C.** Combined dataset of 1-60 age categories (n = 225). (***ntree***, number of trees to grow; **%IncMSE**, increase in mean squared error.)

Supplementary Figure S9

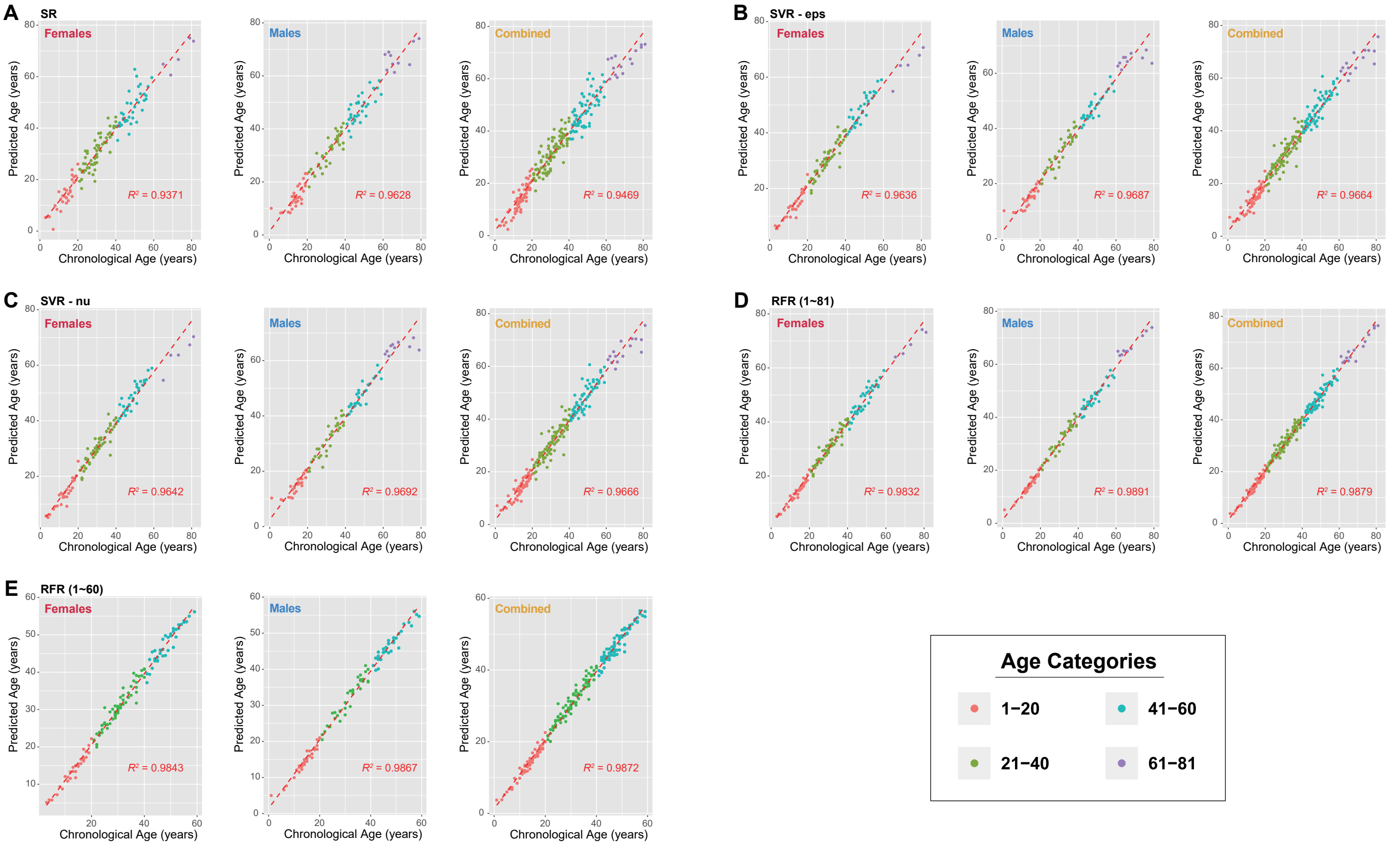

Supplementary Figure S9 Linear relationships between predicted ages and chronological ages in different machine learning models. (R^2, coefficient of determination/goodness of fit)

A. Stepwise Regression (SR) model;

B. Support Vector Regression eps-regression (SVR-eps);

C. Support Vector Regression nu-regression (SVR-nu);

D. Random Forest Regression (RFR) at 1-81 age categories of CHS cohort;

E. Random Forest Regression (RFR) at 1-60 age categories of CHS cohort.
